# Beyond their nutritional value, school meal programs support agricultural and food transition toward sustainability by creating multi-sectoral values in France

**DOI:** 10.3389/fnut.2025.1616375

**Published:** 2025-08-13

**Authors:** Sylvie Avallone, Sophie Nicklaus, Céline Giner, Juliana F. W. Cohen, Stéphane Verguet

**Affiliations:** ^1^MoISA, Univ Montpellier, CIRAD, CIHEAM-IAMM, INRAE, Institut Agro, IRD, Montpellier, France; ^2^Centre des Sciences du Goût et de l’Alimentation, CNRS, INRAE, Institut Agro, Université de Bourgogne, Dijon, France; ^3^Organisation for Economic Cooperation and Development, Paris, France; ^4^NOURISH Lab, Center for Health Innovation, Research, and Policy, Merrimack College, North Andover, MA, United States; ^5^Department of Nutrition, Harvard T.H. Chan School of Public Health, Boston, MA, United States; ^6^Department of Global Health and Population, Harvard T.H. Chan School of Public Health, Boston, MA, United States

**Keywords:** public policies, food education, nutrition, vegetarian meals, climate resilience, economic value, organic farming, equity

## Abstract

The COVID pandemic has highlighted the essential role of school meal programs, not only for education but also for children’s nutrition. In France, school meals are shaped by ambitious policies to ensure their safety and nutritional quality, while promoting sustainable eating practices and awareness of environmental and agricultural challenges. In this article, we used the case study of France to discuss the multi-sectoral value of these programs. The economic value of school meals in France amounts to €8.2 billion annually, of which 2.8 billion are dedicated to food purchases. Since 2022, the EGAlim and Climate and Resilience laws require canteens to offer one vegetarian meal per week and to source at least 50% of sustainable products with positive environmental or social impacts (e.g., certified products, organic farming, and short supply chains). These laws represent a potential support of €1.4 billion for more sustainable agriculture. School canteens also offer a unique opportunity for food education, allowing children to discover new types of food, notably with vegetarian menus. They can contribute to preventing childhood obesity by reducing exposure to ultra-processed foods. Additionally, they play an important role in social inclusion by providing subsidized meals for disadvantaged children. However, disparities in access to canteens persist due to the cost of meals, dietary restrictions or the presence of a parent at home. In conclusion, school meal programs in France generate significant multi-sectoral value in the areas of education, nutrition, agriculture, and social inclusion and support the transition to more sustainable food systems for future generations.

## Background

**FIGURE 1:**
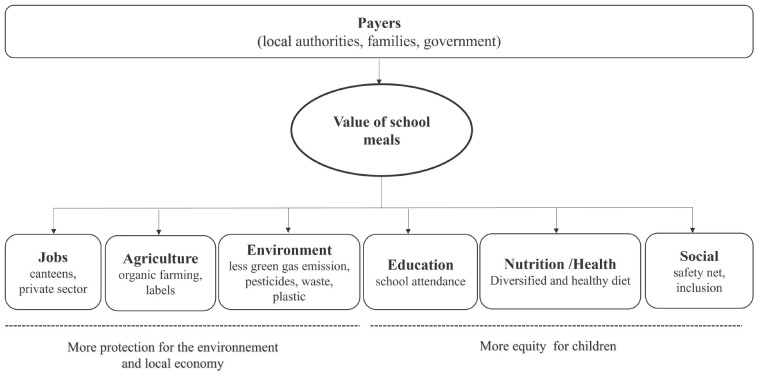
The different dimensions of value and impact associated with the provision of school meals in France.

The COVID pandemic and its ensuing lockdowns made the international community realize the paramount importance of schools, not only in terms of education, but also with regards to the meals that children are able to receive in school canteens every day. Depending on various historical, cultural, organizational and financial contexts, national, regional, or local authorities in charge of implementation have significantly invested in school meal provisions. School feeding programs have demonstrated several effects on education by increasing enrollment and attendance rates, improving nutritional indicators (notably linear growth and micronutrient deficiencies), stimulating local production through institutional procurement, and strengthening household food security (1). Indeed, the potential of school meal programs can largely spill over multiple sectors (2–4). In this article, we take the case study of France to highlight this potential multi-sectoral impact that national school feeding policies can bring (Figure 1) in the field of nutrition but also in other sectors (e.g., agriculture) (5).

**FIGURE 2:**
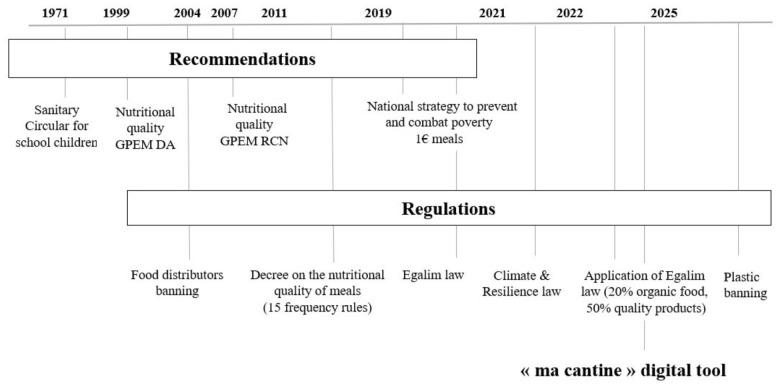
Chronology of national policies for the provision of school meals in France. GPEM/DA: recommendations of the Permanent Study Group on the Markets in Foodstuffs. GPEM-RCN: recommendations of the Market Study Group on Public Catering and Nutrition. “Ma cantine” digital tool: platform for school catering authorities to record their practices. EGAlim law: legislation to strengthen the balance of commercial relations between suppliers and distributors and to increase the supply of organic or quality-labeled products in school canteens (10). Climate and Resilience law: legislation to combat climate change and build resilience by reducing greenhouse gas emissions by 40% by 2030 compared to 1990 levels. Obligation to serve one vegetarian meal a week and to diversify protein sources (11).

First, the French government has a long standing history of establishing regulatory frameworks to guarantee the safety and nutritional quality of meals provided to children at schools (Figure 2) (6, 7). Historically, school meals have been served in restaurants implanted within school buildings, where children can be served while sitting at a table or help themselves at a self-service cafeteria. For example, to ensure a healthy food environment for children, industrial vending machines were banned from schools in 2004 (8). At the menu level, the structure of a school meal includes a starter, a main course served with a side dish, a dairy product, a dessert, and bread. In 2019, school feeding was included in the national strategy to combat poverty with financial support toward meals for children from low-income families in certain areas (i.e., meals provided for one euro or less) (9). Municipalities were financially supported by the French government to make this possible.

Second, the country’s EGAlim (10) and Climate and Resilience (11) laws have provided new support for the agricultural sector and for the transition of food systems toward climate change mitigation. For instance, since January 2022, meals served in school canteens must include (based on the financial purchase value) at least 50% sustainable products with positive environmental externalities (i.e., organic farming, protected geographical indication, quality label, sustainable fishing ecolabel, and traditional specialty guaranteed) or positive social externalities (i.e., supporting small farmers, fair trade, and short supply chains) (10). Menus must also include at least 20% organic products and a weekly vegetarian meal (11). Waste and the use of plastic must be reduced, and students should be better informed about product origin and quality (10, 11). Menus are generally displayed 1 month in advance and organic products are clearly labeled.

## School meals to promote healthy and sustainable eating

The major objective of the French national school feeding policy is to ensure access to healthy, safe and sustainable school meals, while raising children’s awareness of tomorrow’s agricultural, food and environmental challenges. In school canteens, the heritage of French gastronomy (recognized as Intangible Cultural Heritage of Humanity by UNESCO) can be seen in the complex structure of meals (12). Several dish components are served and consumed diachronically (successively in an order defined by social rules) and children eat at table, all together with plates and cutlery.

Such policy has the power to establish a framework facilitating behavior change in youth to address the current challenges observed in the broader population, which often resists nutrition-related public health messages to promote a healthy and balanced diet. For example, only a slight shift toward healthier and more plant-based diets has been observed recently in certain segments of the population (13). In the general population, individual annual meat consumption is relatively stable (around 85 kg per person over 2012–2022) (14), while vegetarians and vegans account for only around 3% of the national population, respectively (15). This highlights the usefulness of public health policies targeting young people which pave the way for the future generations (16) to raise awareness on nutrition issues but also other aspects of sustainability which are typically less prominent in consumers’ minds (17).

To meet the challenges of public health and climate change, French dietary behavior must evolve (18). The school years are an exceptional window of opportunity for educating children about healthy and sustainable eating habits, and school meals provide topical context to set social norms (19). It is at an early age that dietary preferences are established, and they are difficult to modify thereafter (20). School meal programs can therefore play a transformative role, enabling all children to discover new eating practices outside the family environment and supported by peer modeling while socializing and sharing values and norms around food (Table 1) (21). The presence of canteen staff at meal times also makes it possible to educate children about the food they are eating, which can increase their desire to eat it (22).

**TABLE 1:** Summary of the potential positive impacts of school meal provision for healthy and sustainable eating.

Potential positive impacts of school meal provision
**Promotion of food heritage**
- Promotion of gastronomy (meal complex structure, social norms)
- Reconnection to local and seasonal products (tastier, fresher)
**Discovery of healthier eating practices**
- Promotion of diet diversity
- Protection from ultra-processed food products
- Education on healthy eating habits
- Protection from obesity
**Promotion of environmentally sustainable eating practices**
- Organic food and vegetarian meal
- Connection to local farming

Since 2011, a decree has regulated the nutritional quality of school meals served in canteens (7). This decree defines the meal structure, portion size, and 15 service frequencies for different types of dishes over 20 consecutive meals (approximately 4 weeks, or 1 month of school). Certain dishes are favored, while others are limited, without strict prohibitions. For example, fresh fruit or vegetables should be served one meal out of two. Desserts containing more than 15% fat and fried foods should not be offered to children more than three or four times a month.

Compliance with these rules guarantees good nutritional quality and promotes dietary diversity (23). This is supported by research which analyzed 20 consecutive meals in French primary schools and found that adhering to French school food guidelines was associated with higher nutritional quality as assessed by the Mean Adequacy Ratio (MAR), an indicator that estimates the average content of essential nutrients expressed as a percentage of recommended intakes (23). Starters were the main sources of linoleic acid, alpha-linolenic acid, as well as vitamins A and E. The main course provided protein, docosahexaenoic acid (DHA), and vitamins (B_3_, B_6_, B_12_, and D), as well as iron, zinc, iodine, and selenium. Side dishes were the main sources of fiber, vitamin B_9_, potassium, magnesium, and copper while dairy products and desserts contributed to calcium and vitamin C intakes.

Protein source diversification is achieved by including at least one vegetarian meal a week (11). There has also been growing acceptance of vegetarian meals more broadly, with research suggesting that some families are even ready to accept that canteens no longer serve meals with meat or fish on a daily basis (24). Studies have also attempted to evaluate whether this is nutritionally sound and beneficial for the environment. In one study, scenarios were built by mathematical optimization to identify the best trade-off in terms of protein source to limit the environmental impact of the school meals served while preserving nutritional quality (25). One promising lever would be to increase the number of vegetarian meals up to 12 per month (out of 20 meals served) by eliminating red meat and fish. However, the elimination of fish is problematic for the coverage of essential fatty acids. By replacing beef-based meals with plant-based ones, greenhouse gas emissions and land use could be reduced by a third while ensuring good coverage of nutritional needs (25–27).^1^ Research suggests this may also be a strategy that would be acceptable to students. In an experimental study, children’s acceptability of vegetarian and non-vegetarian meals was recorded: over a 9-month period, it was observed that acceptability of vegetarian meals was as high as of non-vegetarian meals (28). Preparing tomorrow’s citizens to vegetalize their plates is a crucial challenge for decarbonizing food systems, and school meals can help meet this challenge by setting new norms. Canteens are encouraged to source products from short supply chains, enabling children to reconnect with their food environment and territory (29). However, the extent to which such a policy deeply impacts children’s attitudes toward foods has not been studied extensively.

Promoting a healthy diet in schools can also be an important lever for obesity prevention and equity. In 2017, overweight and obesity among French children (<17 years old) was 13% and 4%, respectively (30). As in other high-income countries, the prevalence of obesity among children is higher in families with lower education levels (30). In elementary and middle schools, obesity rates are three times less for children from families with higher education levels (30). Highlighting the potentially protective effect of school meals, a cross-sectional study involving primary schools demonstrated that attendance to school canteens was associated with a lower risk of being obese among 5–7-year-olds, regardless of the socio-economic level of the parents (31). School meals can also have a protective role by limiting children’s exposure to ultra-processed foods given the ban on the presence of vending machines in school. Eating nutritionally balanced meals at school may also limit children’s exposure to industrially processed foods available at home (30). Indeed, it has been estimated that when children do not eat in school canteens, their food consumption is composed of two thirds of industrially manufactured products (e.g., desserts and fruit juices) and of only a quarter of homemade products (26).

## School meals to support sustainable agriculture

In France, there are 12,495,000 schoolchildren aged between 5 and 19, and 60% of them eat at school at least four times a week (32). More than 1.1 billion meals are served by 33,000 canteens every year (33). According to the Cour des Comptes (“national court of accounts”), the average full cost per meal was about €7.30 in 2020 (34) including preparation (menu design, preparation, and service), adult supervision of the lunch break (∼€2.70), raw materials (€2.45), fluids (water and energy), and infrastructure investments (equipment and buildings) (Table 2). This cost varies by a factor of three depending on management efficiency and political priorities at the local level (for example, if the municipality wishes to source 100% organic products) (34). The costs of supervising children are relatively high because they are defined by safety standards; the staff involved contributes to educating children about the rules of living together (sharing and respect for food) (34). The average cost of raw materials varies from €1.40 to €2.75 depending on the quality of food supplies (34).

**TABLE 2:** Key features of the school meal programs in France.

Program characteristics	Estimate
Total number of children reached (32)	12,495,000
Coverage (%) (32)	60
Number of school meals served per year	150
Full cost per meal (€) (34)	7.30
Cost of raw material (€) (34)	1.40–2.75

Sources: French Agency for Food, Environmental and Occupational Health and Safety (ANSES) (32) and Cour des comptes (34).

Taking into account the full cost per meal, the number of children eating at canteens, and the number of days of services (approximately 150 days per year), we estimated an annual cost of €8.2 billion. The cost of food purchase would be around €2.8 billion. The EGAlim law (10) requires 50% of these expenditure be oriented toward quality products from more sustainable supply chains generating positive externalities (e.g., organic farming and quality labels). With compliance, the cost of supplying quality products for school meals would constitute a market of €1.4 billion per year, enabling a financial flow toward more sustainable agriculture and diets (Figure 1). In addition, at least 20% must come from organic farming. This law, if fully applied, would support organic farming by around €560 million annually. The economic value focused on sustainable products and sectors constitutes a national lever of action for the French Ministry of Agriculture and Food Sovereignty. As a comparison, this ministry’s annual budget amounted to €6 billion in 2023 and that of the Common Agricultural Policy of the European Union for France was €9 billion (35).

## School meals for social inclusion and equity

School meal programs can fulfill a social role by providing meals at a subsidized price below the full service cost (34). School canteens represent an essential public service for families, particularly when parents work far from home. It allows children from vulnerable families to access a balanced meal at an affordable price (9).

Between 1996 and 2016, canteen attendance rose from 56% to 70% in state schools, but these averages hide major socioeconomic disparities (30, 36). Around 59% of children who do not eat at canteens live in priority education zones (i.e., areas with greater social disadvantage). These zones are defined according to a social index calculated on the basis of the rates of disadvantaged socio-professional categories, scholarship students, students having repeated a year before middle school, and students residing in a priority neighborhood as identified by city policy (37, 38).

A major obstacle to participation in school meals is related to family organization. The presence of an unemployed adult at home (generally the mother) who prepares meals is associated to lower school meal attendance. This is more likely to happen in lower socioeconomic families. When mothers are unemployed, it may be their social role to feed the children (32). Living less than 2 km from the school also allows children to return home easily and encourages non-attendance in canteens (32). Dietary restrictions (e.g., in relation to allergies or religious reasons) and cost of meals are other important factors related to non-attendance.

These disparities in participation limit the full social impact of canteens. To reduce these inequalities, some municipalities subsidize meals by up to €7.70 (e.g., Montpellier and Dijon) translating to an average contribution from families of around €1.70 per meal only (5).

Local authorities can also apply progressive social pricing based on family income and composition. However, 79 and 90% of municipalities with populations of less than 10,000 and 1,000, respectively, use a single meal price, either because of lack of staff or because the number of beneficiaries is too low (39). Since April 2021, to reduce geographical inequalities, the French government provides a €3 aid for each €1 meal served to children in need in primary schools in the smallest municipalities. As a result, 10 million meals at €1 or less have been served nationally. During the 2021–2022 school year, around 123,000 children benefited, and 1,620 municipalities have signed up for this scheme. Yet again, this scheme is only used to a limited extent, undermining its potential impact.

As one example, Saint-Denis is a city with one of the highest poverty rates in France. Indeed, 35% of residents live below the poverty line, which in 2022 was an average income of less than around €1,200 per person per month (40, 41). Since 2021, the city mayor has introduced free meals in nursery schools and in the first three levels of elementary schools. Since this policy’s implementation, attendance by children from disadvantaged families increased by 15%, but without achieving participation by all children. Despite free meals, some disparities according to social origin still persist: dietary restrictions or having an unemployed parent at home limit the participation of some children.

## Conclusion

In France, school meals have been supported by ambitious national policies over several decades, creating value in a number of key sectors. As school meals can increase the time spent at school by disadvantaged children, canteens are a unique place to support equal opportunities and social integration. School meal programs can have a transformative effect. However, further research is needed to assess the benefits in sensitive areas that promote children wellbeing.

Despite a solid regulatory framework, there is still no systematic monitoring and evaluation of the implementation of the laws adopted in France. National implementation of the “ma cantine” digital platform should make it possible to better evaluate the application of nutritional guidelines and the sourcing of “sustainable” products. This should ultimately strengthen the multi-sectoral impact of the school meal program on sustainable development (42). However, as French laws do not impose social pricing or account for cultural and social barriers, school meal programs are currently not able to achieve their full potential in terms of inclusion and equity. Given the potential positive impacts in terms of wellbeing, public health and environmental sustainability, and based on lessons learned from the introduction of social pricing in small localities and the Saint-Denis experiment, mechanisms to expand the attendance of children from disadvantaged families should become a priority of the French school meal policy.

## Data Availability

The original contributions presented in this study are included in this article/supplementary material, further inquiries can be directed to the corresponding author.

## Footnotes

1 Indeed, cattle are ruminants whose digestion produces large amounts of methane (providing as much as 25 times more warming than carbon dioxide); also, beef farming requires large areas of land.
